# Incidental pulmonary embolism in abdominal CT: detection rate and characteristics with artificial intelligence

**DOI:** 10.1093/radadv/umae009

**Published:** 2024-04-30

**Authors:** Peder Wiklund, Koshiar Medson

**Affiliations:** Department of Radiology, Region Halland, Halmstad, 30233, Sweden; Department of Radiology and Functional Imaging, Karolinska University Hospital, Stockholm, 17164, Sweden

**Keywords:** pulmonary embolism, artificial intelligence, retrospective studies, multidetector computed tomography, abdomen

## Abstract

**Background:**

Abdominal CT is a mainstay in the evaluation of abdominal infections, trauma, oncology, and postoperative complications. Pulmonary embolism is a common complication, but there is a risk that these ancillary findings are overlooked. In addition, data on detection rate and characteristics of incidental pulmonary embolism (iPE) on abdominal CT are lacking.

**Purpose:**

The current study compared the period before and after implementing an artificial intelligence (AI) algorithm for iPE detection regarding detection rate and characteristics.

**Material and Methods:**

A retrospective cross-sectional study was performed on abdominal CTs between August 1, 2019, and January 31, 2021 (before AI implementation, 8026 studies) and August 1, 2021, and January 31, 2023 (after AI implementation, 8765 studies). iPE cases were identified through text search and manually confirmed. Study indication and urgency were recorded for iPE patients, and the most proximal iPE level was assessed. A total of 1000 cases after AI implementation were randomly selected and manually reviewed for AI accuracy analysis.

**Results:**

A total of 5876 patients with a mean age of 63.6 ± 17.7 years were included before AI implementation, and 6310 patients with a mean age of 63.2 ± 18.3 years after AI implementation. The iPE detection rate was higher after AI implementation, 0.57% (50/8765 studies) vs 0.12% (10/8026), *P* < .001. The most common study indications were abdominal pain (25%, 15/60 cases) and infection (30%, 18/60 cases). There were no differences in CT pulmonary angiography usage or the most proximal extent of the iPE between the periods before or after AI implementation, *P* > .05. AI identified 46/50 of the reported iPE with 7 AI false-positive cases for a positive predictive value of 87% (95% confidence interval: 75-93%). In the manually reviewed randomly selected subset, iPE prevalence was 1.7% (15/874, 95% confidence interval: 1.0-2.8%) with AI having 40% sensitivity (95% CI, 16-68) and 100% specificity (95% CI, 99.5-100).

**Conclusion:**

Implementing AI for iPE detection and triage increased the iPE detection rate in abdominal CT. The AI sensitivity was moderate, with very few AI false positives.

**Abbreviations**

AI = artificial intelligence, CTPA = CT pulmonary angiography, iPE = incidental pulmonary embolism, PACS = picture archiving and communication system, PE = pulmonary embolism, PPV = positive predictive value

**Summary**

Using an artificial intelligence algorithm to detect and triage incidental pulmonary embolism (iPE) resulted in an increased detection rate of iPE in abdominal CT.

**Key Results**

•  The iPE detection rate was higher after AI implementation, 0.57% (50/8765 studies) vs 0.12% (10/8026), *P* < .001.

•  Most of the iPEs were segmental or more proximal, with no differences before or after AI implementation.

•  The AI algorithm detected 92% of all reported iPEs, with a positive predictive value of 87%.

•  In the manually reviewed cohort, iPE prevalence was 1.7% with an AI sensitivity of 40% and specificity of 100%.

## Introduction

Pulmonary embolism (PE) is a serious condition and the third leading cause of cardiovascular-related deaths.^1^ Although CT pulmonary angiography (CTPA) is the gold standard in PE diagnosis, a substantial proportion of all PEs are discovered incidentally, for example, in conventional chest CT studies.^2^ The symptoms of PE are often nonspecific^3^ and can even present with abdominal pain as the chief complaint.^4^ In addition, PE is a common complication in infection,^5^ cancer,^6^ trauma, and in the postoperative setting.^7^ In abdominal CT, the lower parts of the lungs are visualized, enabling a potential incidental PE (iPE) to be found. In a case series including 18 patients with an iPE present in an abdominal CT, the primary indication was abdominal pain, followed by preexisting conditions such as abdominal abscess, hemorrhage, and postoperative follow-up.^8^

However, because the scans are performed to evaluate abdominal pathology, there is a risk that iPEs are missed if the lung bases are not carefully assessed. The miss rate could be exacerbated by the scans commonly done in the portal venous phase, leading to lower contrast enhancement in the pulmonary arteries compared with a CTPA or a late arterial phase chest CT. In chest CTs, previous studies have reported that 32% to 79% of iPEs were missed in the original interpretation; a substantial proportion of iPEs are likely missed in abdominal CT as well.^9–13^

Artificial intelligence (AI) algorithms have shown the potential to improve iPE and PE detection. Previous retrospective and prospective studies have shown that an AI algorithm had high accuracy in detecting PE and iPE in CTPA and chest CT.^13–15^ Previous studies in patients with cancer have shown that the iPE detection rate in chest CT increased from 0.8% to 2.5% after AI implementation^16^ and that the prospective use of AI was associated with a reduced rate of missed iPE, from 44.8% to 2.6% after AI implementation.^17^ Regarding abdominal CT, a retrospective study evaluating an AI algorithm for iPE detection showed a prevalence of 0.9%, with 39% missed in the original interpretation.^18^ However, no studies are reporting on the clinical use of AI in abdominal CT.

The study aimed to assess the detection rate of iPE in abdominal CT, assess iPE characteristics, and compare the periods before and after implementing an AI algorithm for iPE detection and triage.

## Materials and methods

### Design

A multicenter retrospective cross-sectional study was conducted in Halland Hospital, Region Halland, Sweden, comprising 3 study hospitals: Halmstad, Varberg, and Kungsbacka. The Swedish Ethical Review Authority approved the study protocol. Informed consent was waived because of the retrospective nature of the study.

### Patients

All contrast-enhanced abdominal CT studies on patients of at least 18 years of age were included between August 1, 2019, and January 31, 2021 (before AI implementation) or between August 1, 2021, and January 31, 2023 (after AI implementation), ordered from the emergency department and in the inpatient and outpatient settings. All radiology reports were automatically searched for mentions of “embolism,” “pulmonary embolism,” or “filling defects” and were then manually confirmed to be either positive or negative for iPE. Patients with iPE were excluded if the iPE was already reported in a prior study or if a chest CT or CTPA was performed at the same time as the abdominal CT.

### CT scan parameters

All studies were performed on a 64-slice multidetector CT scanner (Revolution CT, GE Healthcare) with 120 kVp and mAs chosen using automatic tube current modulation. Collimation was 64 × 0.625 mm. The 0.625-mm images were always sent to the picture archiving and communication system (PACS), with additional reconstructed 5-mm images in the transverse, coronal, and sagittal planes. Patients received 500 mg iodine/kg for abdominal CT, up to a maximum dose corresponding to a body weight of 80 kg. The scans used automatic bolus tracking with an attenuation threshold in the descending aorta of 100 HU and a scan delay of 63 seconds for the abdominal scan for a portal venous phase. In cases of an estimated glomerular filtration rate <45 mL/min, a reduced contrast media volume was considered on a case-by-case basis after reviewing the indication of the examination. The cranial extent of the scan was defined as above the diaphragm, based on CT scout images.

### AI solution and clinical workflow

The AI solution is a commercially available cloud-based solution for iPE and PE detection and triage (Aidoc BriefCase, Aidoc Medical). Details concerning training and validation have been previously published.^15^ In brief, the AI algorithms were trained and validated on tens of thousands of CT examinations acquired on a diverse range of CT scanners optimized for study-level classification. There are separate AI algorithms for analyzing CTPA studies and late arterial and portal venous phase studies. Studies are automatically uploaded to the cloud, where an image-based analysis is performed to automatically assign which AI algorithm is suitable to analyze the case.

AI results were presented in a widget separate from the PACS, and all AI results were immediately made available to the reporting radiologist in the regular workflow. There was no policy regarding the need for further evaluation with CTPA in cases of a detected iPE or whether a CTPA was recommended or not was at the discretion of the reporting radiologist.

### Reference standard for iPE detection

A retrospective review of all reported iPE and all AI-positive cases was conducted by the authors, a cardiothoracic radiologist with 7 years of experience and a general radiologist with 10 years of experience. Images were reviewed in the PACS (Sectra, Sectra AB). iPEs were grouped into 4 groups: subsegmental, segmental, lobar, or central iPE, with final categorization by consensus. Study indication, urgency level, and patient characteristics were reviewed in the PACS.

To more fully evaluate the AI accuracy parameters, 1000 cases were randomly selected from the post-AI period and were reviewed for the presence of iPE by the general radiologist reader, blinded to AI results and clinical information. HU values were measured in the most proximal part of the pulmonary arteries that were visualized for each scan.

### Statistics

Standard descriptive statistics were used on a per-study level to compare iPE detection rate and characteristics. The chi-square test was used for comparing categorical variables. The exact Clopper-Pearson interval was used for calculating CIs for proportions. Categorical data are presented as percentages, and continuous data as the mean ±1 SD. *P* < .05 was considered statistically significant. SPSS Statistics for Windows, version 27 (IBM Corp.), was used for all analyses.

## Results

### Cohort description

**Figure 1.**
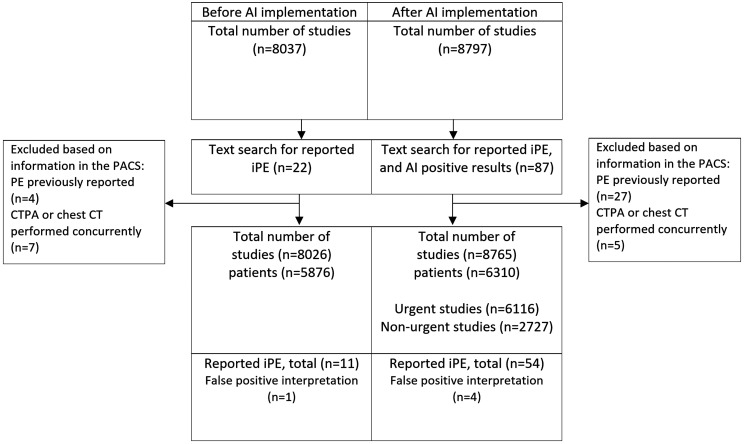
Flowchart of the inclusion process. PE = pulmonary embolism; iPE = incidental pulmonary embolism.

A total of 8026 examinations were included before AI implementation and 8765 examinations after AI implementation. The inclusion and exclusion process is shown in Figure 1; the baseline characteristics of the study population are shown in Table 1. Before AI implementation, the mean age of the included patients was 63.6 ± 17.7 years, with 53.6% being female. After AI implementation, the mean age was 63.2 ± 18.3 years, with 57% being female. Of the 54 reported iPE cases after AI implementation, 2 AI-positive and 2 AI-negative cases were deemed to be false positive after either a CTPA (1 case) or during the retrospective image review (3 cases) compared with 1 of 11 cases before AI implementation (after a CTPA).

**Table 1.** Baseline characteristics of the included population were stratified before and after AI implementation.

	Before AI implementation	After AI implementation	*P*
Number of patients (number of studies)	5876 (8026)	6310 (8765)	
Age, years	63.6 ± 17.7	63.2 ± 18.3	.23
Women	53.6%	57.0%	<.001
iPE detection rate (n/total studies)	0.12% (10/8026)	0.57% (50/8765)	<.001
*iPE patient characteristics, n*	10	50	
Age	63 ± 18	71 ± 15	.15
Sex (women)	40%	60%	.24
*Study priority*			.86
Urgent	90%	88%	
Nonurgent	10%	12%	
*Primary study indication*			.98
Abdominal pain	30%	24%	
Infection	20%	32%	
Bowel obstruction	10%	12%	
Oncologic	10%	8%	
Postoperative complication	10%	10%	
Other	20%	14%	

AI = artificial intelligence; iPE = incidental pulmonary embolism.

### iPE detection rate and characteristics

**Figure 2.**
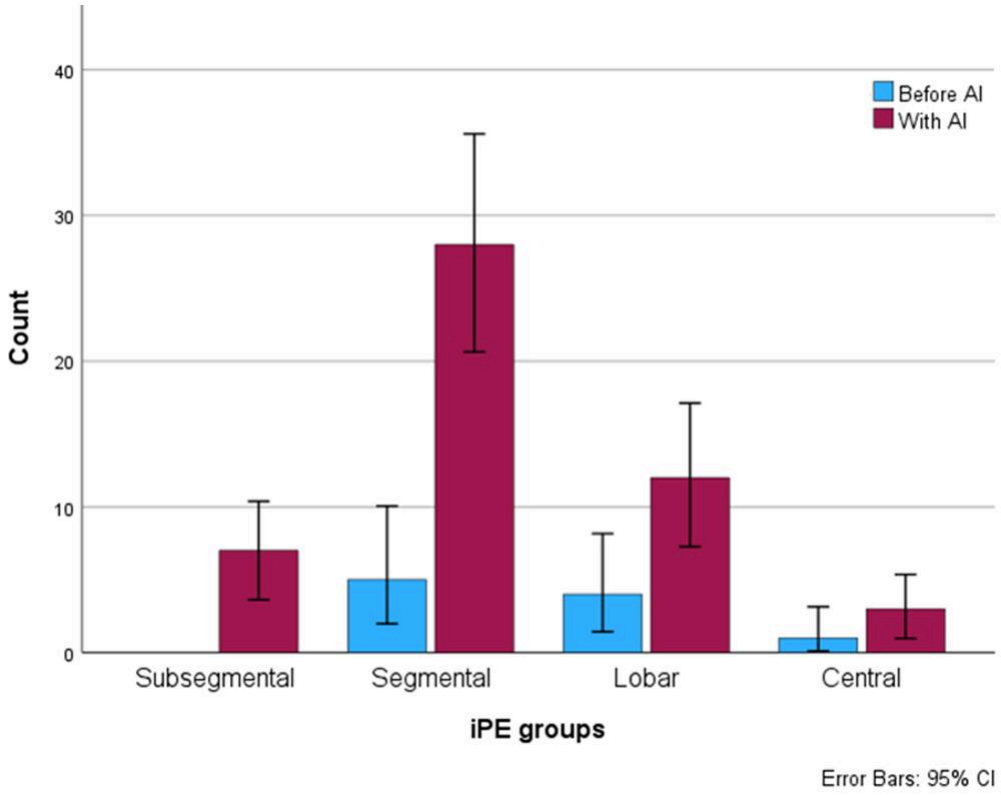
Distribution of the most proximal extent of the iPE. iPE = incidental pulmonary embolism.

The iPE detection rate was higher after AI implementation, 0.57% (50/8765 studies) vs 0.12% (10/8026), *P* < .001. The iPE detection rate was 0.73% (44/6049) for urgent studies and 0.22% (6/2716) for nonurgent studies after AI implementation, whereas stratification of the studies before AI implementation was not possible because of a change in PACS. Baseline characteristics and study indications of the patients with iPE are presented in Table 1, with the most common primary indications for the CT study being infection (30%, 18/60 cases) or abdominal pain (25%, 15/60 cases). There was no evidence of a difference in the most proximal extent of the iPE between the periods before or after AI implementation (*P* = .48). Before AI implementation, the most proximal extent of the iPEs were segmental in 50% and lobar or more proximal in 50%; after AI implementation, the iPEs were subsegmental in 14%, segmental in 56% and lobar or more proximal in 30%, as shown in Figure 2.

After AI implementation, CTPA was performed in 20% (10/50) of cases for confirmation of PE diagnosis and in 18% (9/50) for evaluation of total PE extent, compared with 30% (3/10) and 20% (2/10), respectively, before AI implementation, with no difference in CTPA usage between the periods (*P* = .74). In the 24 patients with a CTPA, the CTPA showed additional emboli in 70.8% (17/24) compared with the initial abdominal CT.

### AI accuracy

**Figure 3.**
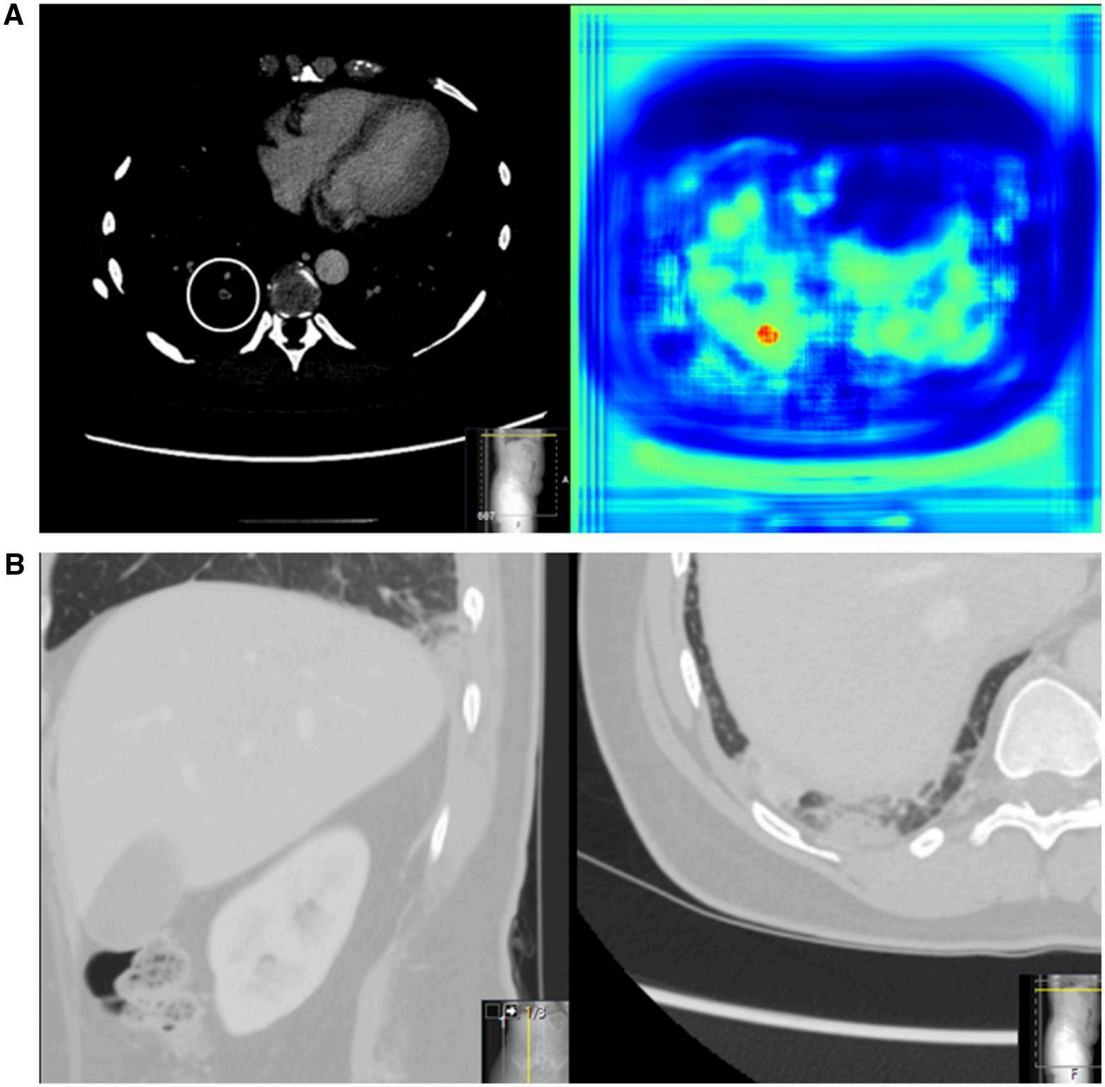
iPE and lung infarction in a middle-aged female patient with right-sided flank pain. There were no abnormalities in the abdomen, and no explanation was found other than a primary PE. (A) iPE in a posterior segmental artery in the right lower lobe, visualized in the axial plane in a standard contrast-enhanced abdominal CT. The AI heatmap is shown to the right. (B) Pulmonary opacification in the basal parts of the right lower lobe was interpreted as a lung infarction, visualized in the axial and sagittal plane. AI = artificial intelligence; iPE = incidental pulmonary embolism; PE = pulmonary embolism.

**Figure 4.**
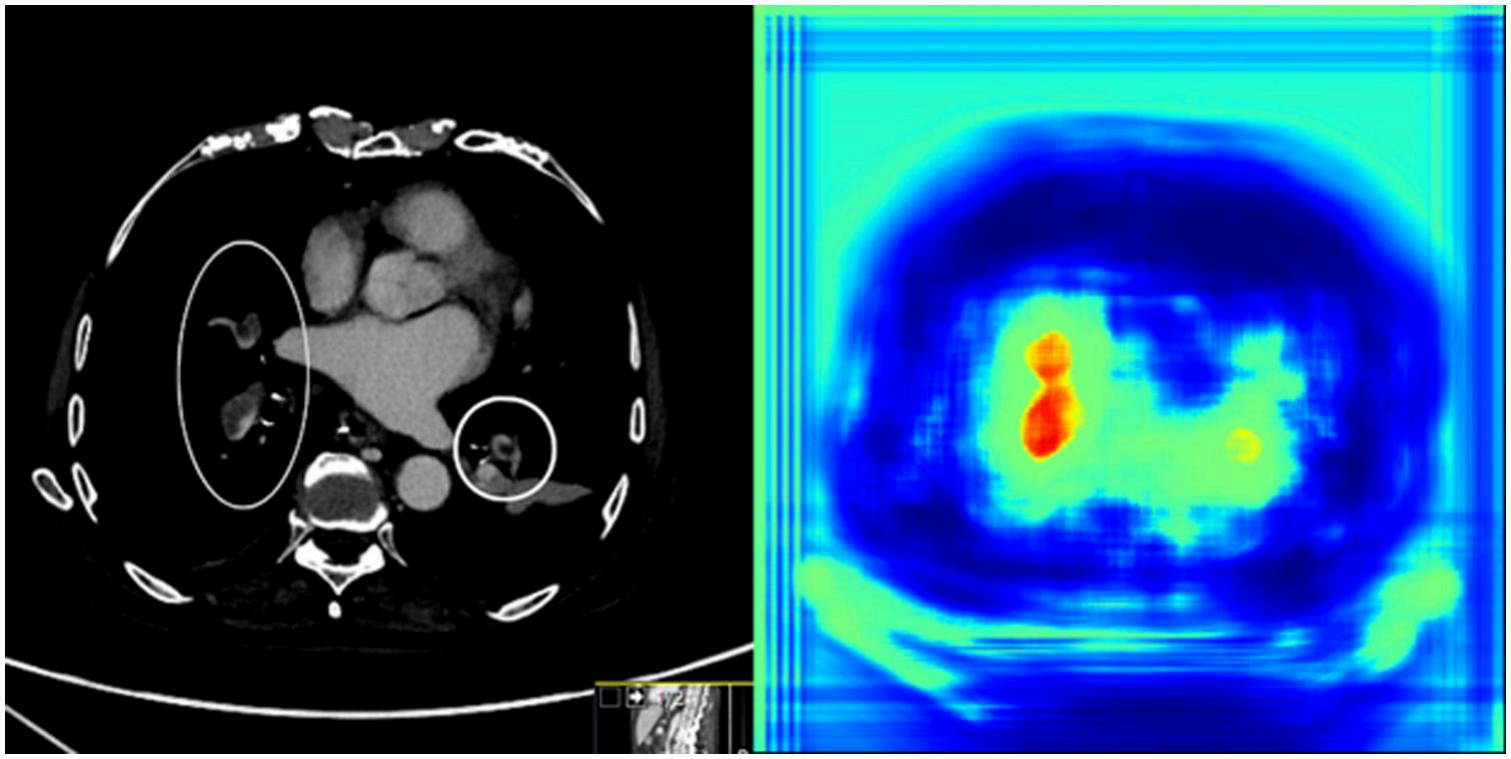
Massive iPE in an elderly female patient with abdominal pain after colonoscopic stenting. iPEs are present in the lobar arteries to the right middle and lower lobe and segmental arteries to the left lower lobe, visualized in the axial plane in a standard contrast-enhanced abdominal CT. The AI heatmap is shown to the right. AI = artificial intelligence; iPE = incidental pulmonary embolism.

**Figure 5.**
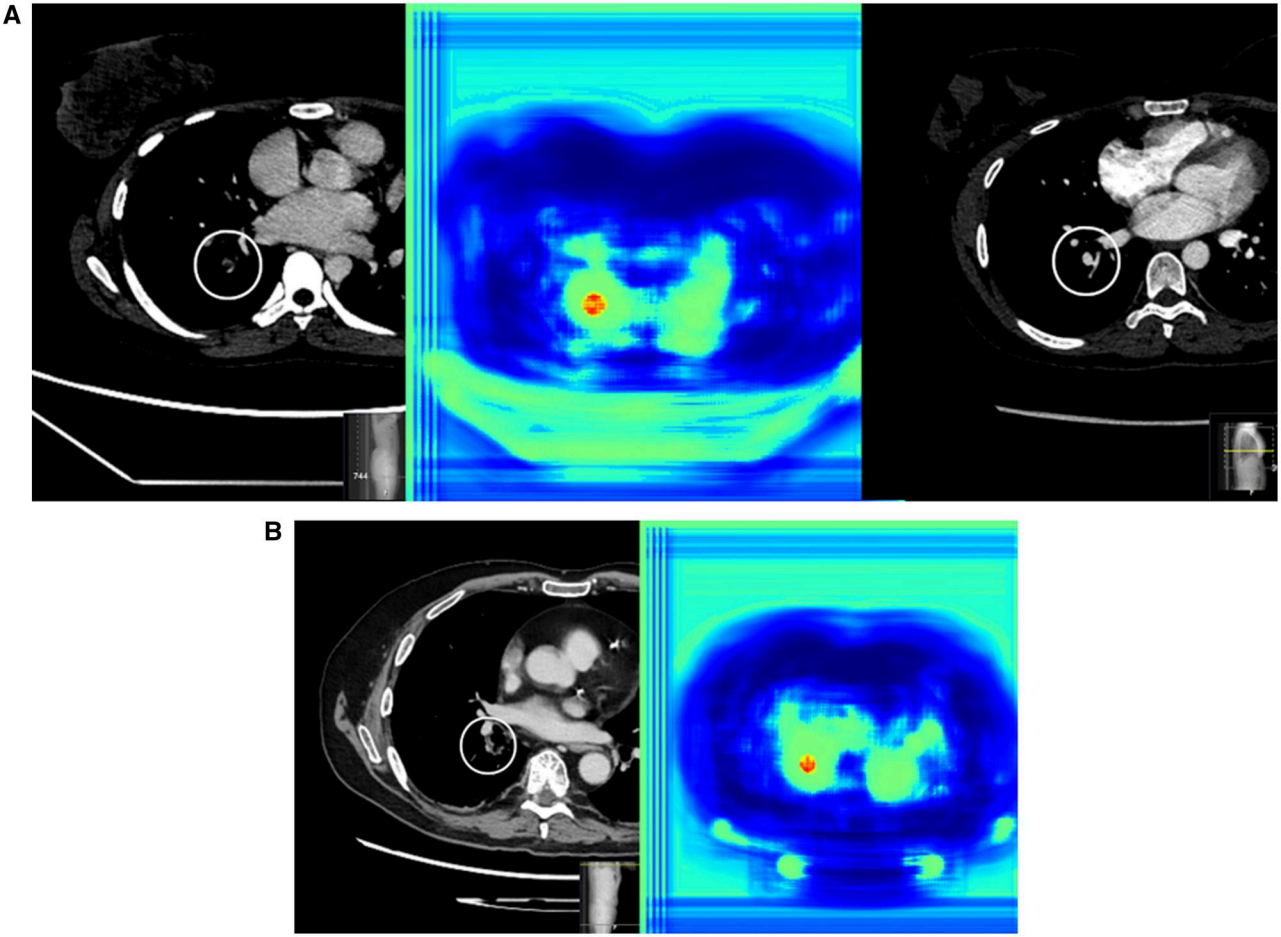
Cases of AI false positives in standard contrast-enhanced abdominal CT. (A) A young woman with right upper quadrant abdominal pain. AI false positive for PE, the finding is visualized in the axial plane to the left, with the AI heatmap in the middle. Initially reported as true positive, but confirmatory CTPA (to the right) showed that it was a false-positive finding resulting from streak artifacts. (B) An older man with facial trauma and right abdominal pain after a fall, AI false positive for PE. The finding is visualized in the axial plane to the left, with the AI heatmap to the right. The finding corresponds to slightly prominent peribronchovascular interstitium. AI = artificial intelligence; CTPA = CT pulmonary angiography; PE = pulmonary embolism.

**Figure 6.**
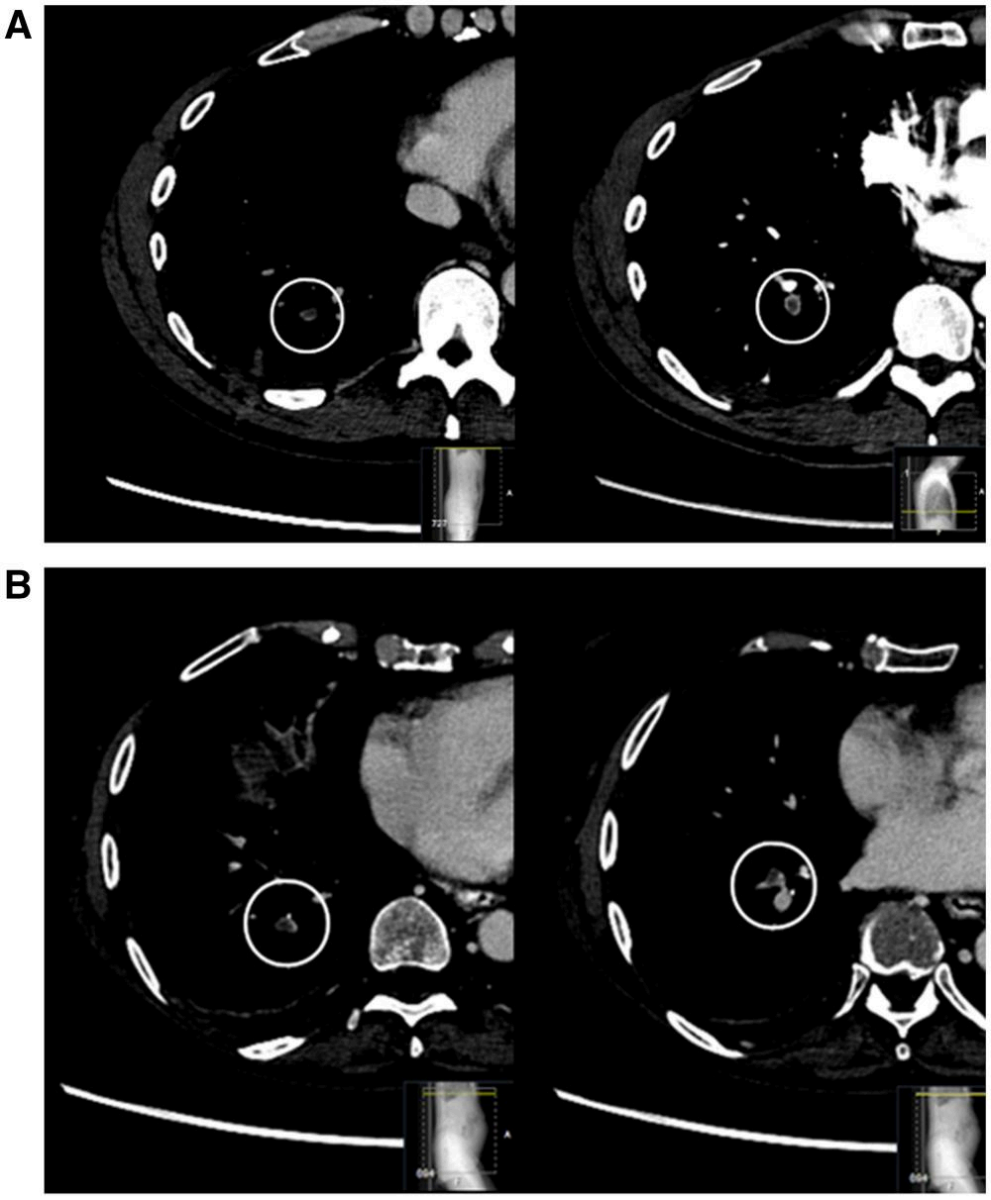
Cases of reported iPE that were AI false negative in standard contrast-enhanced abdominal CT. (A) A middle-aged man with upper right quadrant abdominal pain and elevated C-reactive protein levels, AI false negative for PE. A filling defect in a subsegmental artery in the right lower lobe was reported as suspicion of PE, shown to the left. Confirmatory CTPA was recommended, showing that the PE in the right lower lobe was segmental (shown to the right), with additional PE in the middle lobe and the left lung (not shown). (B) An older women with pancreatic cancer and metastases, presenting with abdominal distension and nausea. AI false negative for PE. Abdominal CT shows tumor progression and PE in segmental and subsegmental arteries, visualized in the axial plane. AI = artificial intelligence; CTPA = CT pulmonary angiography; PE = pulmonary embolism.

AI identified 46/50 of the reported iPE with 7 AI false-positive cases for a positive predictive value (PPV) of 86.8% (95% CI, 75-93). Examples of AI-detected iPE are presented in Figures 3 and 4. Four of the reported iPE cases were AI false negatives. Cases of AI false negatives and false positives are shown in Figures 5 and 6. Although the presented false-positive cases could be attributed to streak artifacts and slightly prominent peribronchovascular interstitium, there was no apparent cause for the AI false-negative cases. Of the 7 AI false-positive cases, 3 were classified as AI true positive in the retrospective image review. In 1 case, there was a single segmental iPE; in 2 cases, there were single subsegmental iPEs.

In the manual review of the 1000 randomly selected cases, 126 cases were excluded based on the examination not having been performed (*n* = 110), erroneously classified protocols (*n* = 12), or no parts of the lungs being imaged (*n* = 4). In 874 cases, 15 iPE cases were found for an iPE prevalence of 1.7% (95% CI, 1.0-2.8). AI correctly identified 40% (6/15 iPE cases), with no false-positive findings for 40% sensitivity (95% CI, 16-68), 100% specificity (95% CI, 99.5-100), 100% PPV, and 99% negative predictive value. Although 5 of 6 AI true-positive cases were reported in the radiology report, none of the AI false-negative cases was reported. Attenuation in the most proximal visualized pulmonary vessels were 187 ± 89 HU for iPE-positive cases and 140 ± 26 for iPE-negative cases (*P* < .001).

## Discussion

Incidental PE on abdominal CTs can go undetected because the pulmonary arteries might not be carefully assessed and the contrast phase is not optimized for the pulmonary arteries. In the current study, including 3 radiology departments in Region Halland, the iPE detection rate on abdominal CT was significantly higher after implementing an AI algorithm for iPE detection and triage: 0.57% vs 0.12%. There were no differences in patient or iPE characteristics, and there was a similar usage of CTPA, with the vast majority of the iPE being segmental or more proximal. The AI detected 92% of all reported cases with a PPV of 87%. In the randomly selected sample for AI accuracy analysis, the iPE prevalence was 1.7% and AI had 40% sensitivity and 100% specificity with no false-positive findings. Thus, the AI algorithm improved iPE detection rates with very few false-positive cases.

The AI sensitivity for iPE in abdominal CT was lower than in previous studies using late arterial and portal venous phase chest CT, whereas the PPV was comparably high.^13^^,^^17^ One reason could be that the PE is visible in fewer slices and that a cropped cranial PE extent could negatively affect the detection rate. However, no conclusions can be made because the AI output is binary and no information is presented regarding image quality or if parts of the scans are excluded from analysis.

There are very few previous studies regarding both iPE prevalence and AI in abdominal CT. Wildman-Tobriner et al. used AI for iPE detection and natural language processing for classifying reports, showing an iPE prevalence of 0.9% (49/5515), of which 39% were unreported.^18^ The AI algorithm had a much higher number of AI false positives, with a PPV of only 14%, compared with a PPV of 87% in the present study. As a false positive iPE diagnosis could expose patients to unnecessary anticoagulant treatment, the risk of automation bias must be considered. In the present study, 2 reported AI-positive iPE cases were reclassified as false positives after either a CTPA or the retrospective image review. False-positive cases also occurred in 2 AI-negative cases and 1 case before AI implementation, and in 3 cases, AI true-positive findings were erroneously classified as false positives. Although the detection rate was 0.57% after AI implementation, the iPE prevalence in the manually reviewed subset was 1.7%, with 9 of 15 cases both AI false negative and not reported in the radiology report. Thus, even with the aid of the current AI algorithm, this highlights the need to carefully assess the pulmonary arteries before confirming or rejecting a PE diagnosis.

Patients with iPE detected in abdominal CT are a heterogeneous group because thromboembolic complications occur in a multitude of underlying conditions and are associated with adverse outcomes (eg, in the postoperative setting^19^^,^^20^ in cancer^21^ and infections^22^). An abdominal disorder could mask the symptoms of an acute PE, and PE-related complaints could be attributed to an abdominal disorder, as exemplified in Figure 4 and previous case reports.^4^^,^^23^^,^^24^ Most of the iPE in the current study were segmental or more proximal. When CTPA was performed, 70% showed additional emboli compared with the initial abdominal CT. Current guidelines recommend offering anticoagulant therapy for segmental or more proximal emboli, whereas isolated subsegmental iPE treatment should be offered on a case-by-case basis.^25^^,^^26^ Whether the reporting radiologist should recommend further evaluation with CTPA depends on several factors, including the clinical situation, the extent and multiplicity of the PE, and confidence in the PE diagnosis. CTPA should be considered when the PE diagnosis is uncertain, and CTPA to determine total PE extent should be considered for image-based risk stratification of the PE.^26^ A discussion with the referring physician is often valuable in both the inpatient and outpatient settings.

## Limitations

Because the included CT studies were automatically collected and stratified based on PACS coding and text search, erroneous inclusions were unavoidable, causing an underestimation of the reported detection rate. In the randomly selected subset of cases manually reviewed to evaluate AI accuracy, 12.6% of cases were excluded for various reasons. It was not possible to evaluate how many correctly performed scans could not be analyzed by AI. In addition, as attenuation in the portal venous phase is lower compared with a CTPA or late arterial phase, iPE prevalence in subsegmental arteries is likely underestimated. Receiver operating characteristic analysis was not possible because AI output was binary, using a fixed threshold for classifying studies as positive or negative.

## Conclusion

Implementing an AI for iPE detection and triage increased the detection rate of iPE in abdominal CT, with the vast majority of the iPE being segmental or more proximal. The AI sensitivity was moderate, with very few false positives.

## Supplementary Material

umae009_Supplementary_Data
